# Multi-beam X-ray ptychography using coded probes for rapid non-destructive high resolution imaging of extended samples

**DOI:** 10.1038/s41598-022-09466-5

**Published:** 2022-04-13

**Authors:** Mikhail Lyubomirskiy, Felix Wittwer, Maik Kahnt, Frieder Koch, Adam Kubec, Ken Vidar Falch, Jan Garrevoet, Martin Seyrich, Christian David, Christian G. Schroer

**Affiliations:** 1 Center for X-ray and Nano Science CXNS, Deutsches Elektronen-Synchrotron DESY, Notkestr. 85, 22607 Hamburg, Germany; 2 Department Physik, Universität Hamburg, Luruper Chaussee 149, 22761 Hamburg, Germany; 3 Present Address: NERSC, Lawrence Berkeley National Laboratory, Berkeley, CA 94720 USA; 4 MAX IV Laboratory, Lund University, Box 118, 221 00 Lund, Sweden; 5 Paul-Scherrer-Institut (PSI), Forschungsstr. 111, 5232 Villigen, Switzerland; 6 GSI Helmholtzzentrum für Schwerionenforschung GmbH, Planckstr. 1, 64291 Darmstadt, Germany; 7 XRnanotech GmbH, Forschungsstr. 111,ODRA 117, 5232 Villigen, Switzerland; 8 Helmholtz Imaging Platform, Deutsches Elektronen-Synchrotron DESY, Notkestr. 85, 22607 Hamburg, Germany

**Keywords:** X-rays, Microscopy, Nanoscience and technology, Imaging techniques

## Abstract

Imaging large areas of a sample non-destructively and with high resolution is of great interest for both science and industry. For scanning coherent X-ray diffraction microscopy, i. e., ptychography, the achievable scan area at a given spatial resolution is limited by the coherent photon flux of modern X-ray sources. Multibeam X-ray ptychography can improve the scanning speed by scanning the sample with several parallel mutually incoherent beams, e. g., generated by illuminating multiple focusing optics in parallel by a partially coherent beam. The main difficulty with this scheme is the robust separation of the superimposed signals from the different beams, especially when the beams and the illuminated sample areas are quite similar. We overcome this difficulty by encoding each of the probing beams with its own X-ray phase plate. This helps the algorithm to robustly reconstruct the multibeam data. We compare the coded multibeam scans to uncoded multibeam and single beam scans, demonstrating the enhanced performance on a microchip sample with regular and repeating structures.

## Introduction

X-ray ptychography has developed into a robust imaging technique with applications in many fields of science, allowing to visualize complex processes in nano-structured materials with high sensitivity and spatial resolutions down to below 10 nm^1–4^. One of the remaining challenges in ptychography is the fast scanning of larger sample areas in a shorter time while preserving the highest resolution in the reconstructed image^5–7^. One approach to tackle this challenge is to increase the rate at which data is taken, for example by minimizing overheads and continuously (fly-)scanning. With this approach of speeding up the data rates of conventional ptychography, the available coherent flux will eventually pose the upper limit on how quickly data can be recorded while preserving the resolution in the reconstruction. Since the coherent fraction of the monochromatic beam at third-generation synchrotron radiation sources is in the one percent range, most of the beam remains unused in this technique. This limits the field of view and resolution in ptychography, typically trading the one off against the other. Experiments that require both, are typically time consuming and thus sometimes not feasible.

For instance, to understand reactions in heterogeneous catalysis the chemistry of relevant structures on the single-digit nano-scale need to be followed under working conditions over time and extended regions inside a reactor^8,9^. Here, besides a high spatial resolution^10^ also chemical sensitivity^11,12^ is needed. Both require high fluence, currently limiting the field of view. However, such experiments are essential to derive structure-activity relationships, leading to the design of more sustainable and efficient materials for purposes such as emission control, synthesis of industrial-scale chemical products, and conversion or storage of energy-related chemicals. Decreasing the time needed to scan larger representative samples will enable the tracking of dynamic changes in heterogeneous catalysts as a function of the environment and potentially allowing proper kinetic studies such as reactor rates or activity^9^. Similar requirements are found for other projects, such as the RAVEN program of IARPA^13^, where millimeter-scale microchips need to be imaged with resolutions down to the transistor level.

One way to overcome the current limitations is to increase the brightness of the source, which is pursued by most synchrotron radiation sources around the world and will lead to significant advances in the next decade^14–16^. Another complementary way is to exploit the multibeam approach in ptychography^17^. In this scheme, the object is scanned simultaneously with several spatially separated mutually incoherent probes. This parallel acquisition of several parts of the sample increases the field of view while preserving the fluence and thus the sensitivity and resolution.

Multibeam ptychography was demonstrated with visible light^17,18^ and with X-rays using spatially separated slits^19^, Fresnel zone plates^20^ and refractive lenses^21^. Best use of the incident X-rays can be made if the optics that generate the probes cover a large area of the incident beam. This was demonstrated using an array of 3D printed refractive lenses^22^ that captured 92 % of the beam irradiating the optics, creating up to six spatially separated probes^21^. In this experiment, the probes were all very similar and arranged in an array with a distance of 46 μm, which is small compared to the detector pixel size of 75 μm. This makes it difficult in the reconstruction to disentangle the diffraction patterns created by the different probes, in particular when the sample is weakly scattering. As a result, artifacts can appear, such as superimposed ghost images of regions scanned by neighboring probes.

This ambiguity in the inverse problem can be avoided by scanning the sample with a set of very different probes. In this work, we present multibeam ptychography with coded probes, which allows us to disentangle the contributions from multiple probes robustly, accelerating the ptychographic reconstruction and utilizing large fraction of the beam irradiating the optics. The wavefront of each probe was individually adjusted by tailor-made phase plates^23,24^ that were 3D printed together with the focusing optics. This results in highly diverse coded probes and significantly improves the reconstruction quality and speed compared to the same measurement but with similar probes. This approach solves the problem of probe ambiguity in multibeam ptychography and makes the technique reliably applicable in practice.

## Results

**Figure 1:**
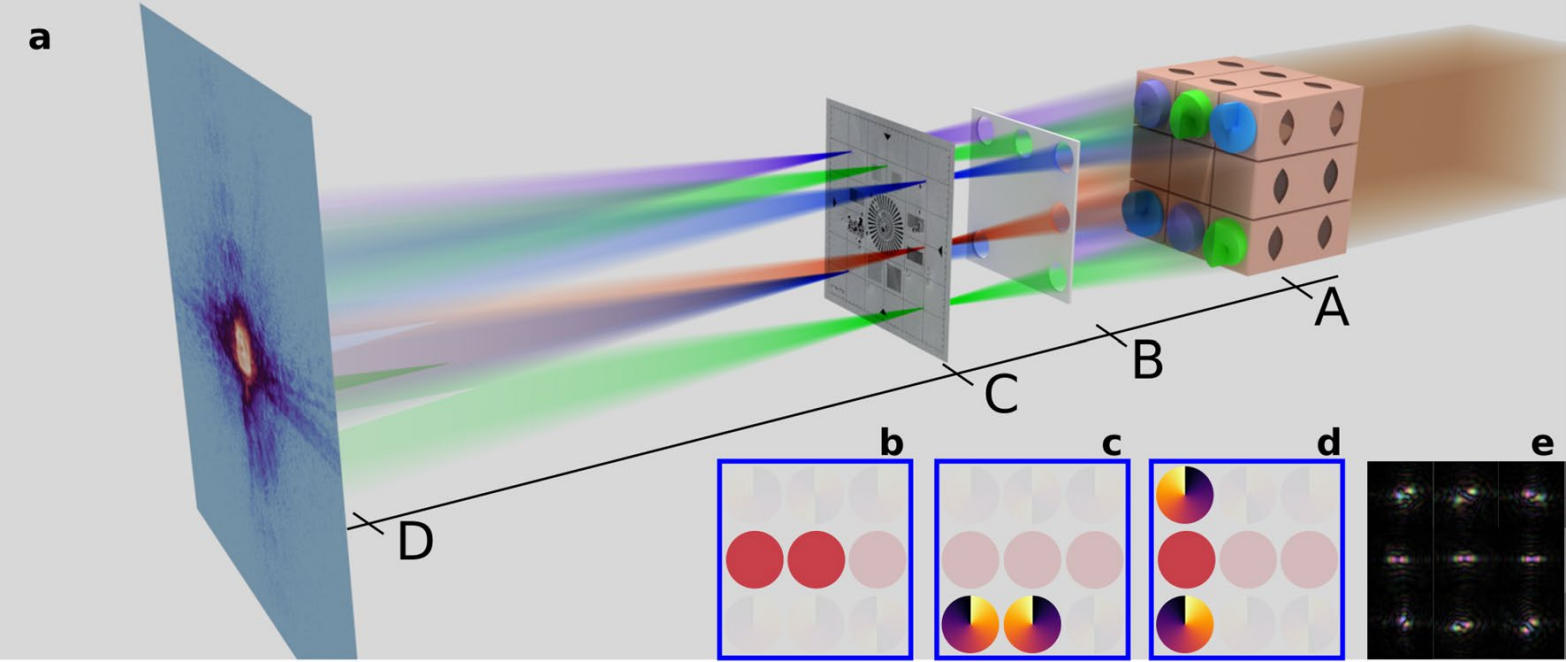
(**a**) Experimental setup with the lens array (A), the pinhole array (B), the sample (C) and the detector (D). (**b**) Lens combination generating two uncoded probes. (**c**) Lens combination generating two coded probes. (**d**) Lens combination generating three coded probes. (**e**) All nine probes, reconstructed in the sample plane at 7 keV.

The X-ray-optical experiments were carried out at the microprobe end-station of beamline P06^25^ of the synchrotron radiation source PETRA III at DESY in Hamburg. The experimental scheme is illustrated in Fig. 1a. We used an array of 3D-printed compound refractive lenses^22^ to create multiple probes (photon energy range: 7–9 keV). As our primary goal was to show a practical solution to the problem of disentangling the scattered signal from neighboring beams, for better reader understanding, we designed the lens array to use vertical and horizontal arrangements with only two and three beams. Some of these lens stacks were equipped with phase plates to create vortex beams with topological charges of + 1, − 1, or + 2.

Slits (not shown) in front of the lens array ((A) in Fig. 1a) and a pinhole array ((B) in Fig. 1a) between the lenses and the sample ((C) in Fig. 1a) were used to select various combinations of lenses, defining the arrangement, number and shape of different probes. A Siemens star resolution chart made by NTT-AT (model XRESO-50HC) with smallest features of 50 nm was imaged to prove the performance enhancement. A microchip manufactured by Infineon Technologies AG was imaged to validate the technique with a real-life sample.

### Method verification

To investigate the influence of coded probes as compared to similar ones with the most basic case of multibeam ptychography – a two-beam case, we recorded a two-beam ptychogram of a sample (Siemens star) with similar probes and one with coded probes with topological charges of + 1 and − 1, respectively. The same region of the Siemens star was scanned during both scans, using identical scan parameters with probes arranged horizontally (Fig. 1b and 1c.) The size of the scan area was chosen to have an overlap area of 10 μm × 4 μm (*v* × *h*), which was scanned by both probes. The total area covered by either probe during the scan was 10 μm × 50 μm (*v* × *h*). The scans were recorded in a step-settle-expose mode and took around 15 minutes each, including time for the movement and settling of the scanning stage. Scanning the object with the same fluence using a single beam would have taken 1.9 times longer. Afterwards, both scans were reconstructed using the same algorithm.

**Figure 2:**
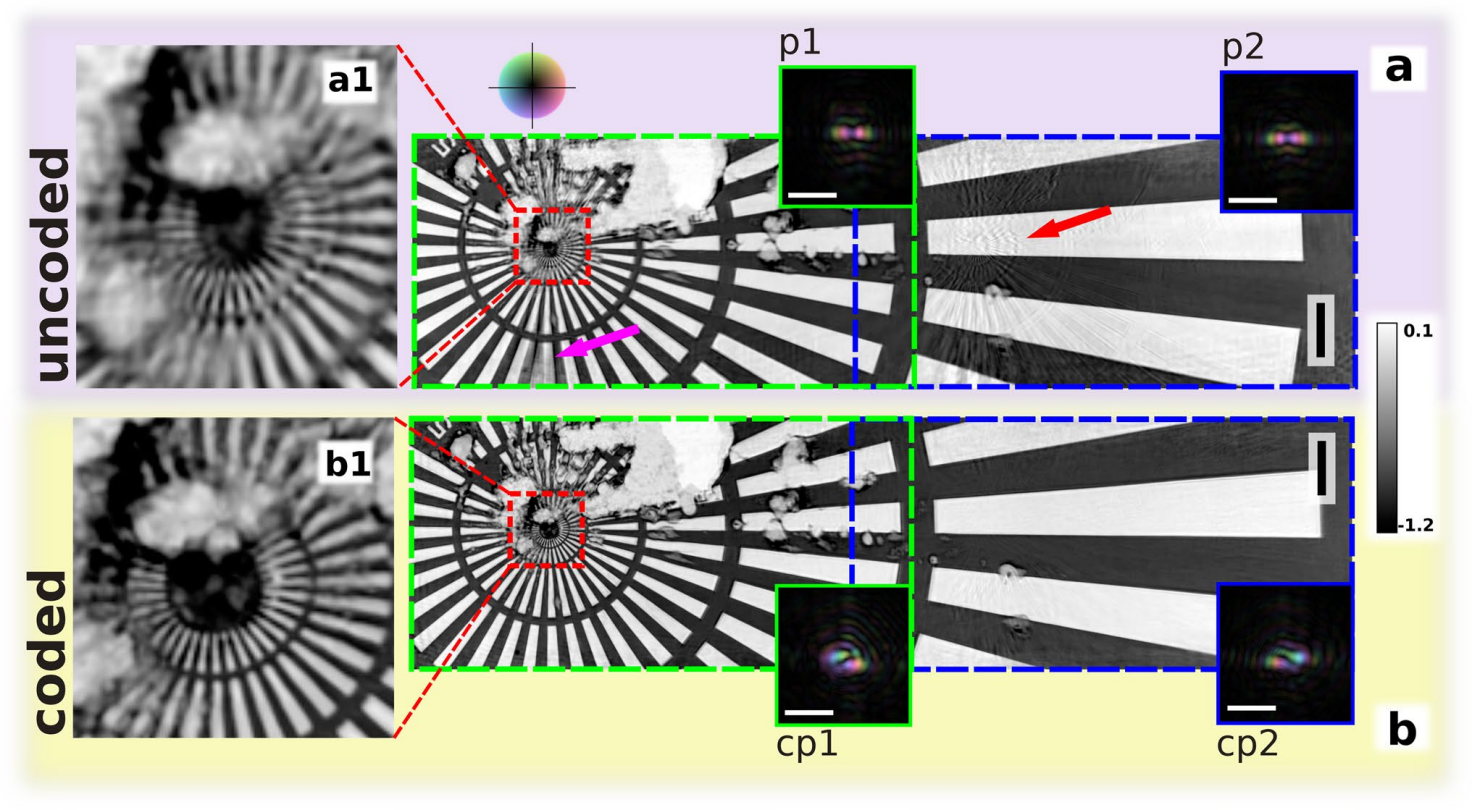
Reconstructed images of a Siemens star test sample and corresponding probes. Red and purple arrows show reconstruction defects. Blue/green dashed rectangles show which probe scanned which region of the sample: (**a**) uncoded probe case with the probes shown in (p1) and (p2), (**a1**) is the magnified center of the test pattern; (**b**) coded probe case, with the probes shown in (cp1) and (cp2), (**b1**) is the magnified center of the test pattern. The vertical scale bars represent 3 μm, the horizontal scale bars represent 4 μm. The gray scale indicates the sample phase shift in radians. Phases of the probes are color-coded.

Figure 2 shows the reconstructed phase images of the Siemens star after 1000 iterations together with the corresponding complex wave fields of the probes used to illuminate the sample. In the first case using identical probes, the presence of large scale image artifacts on the object is evident (see Fig. 2a): the copy image of the center of the Siemens star located on the right side (marked with the red arrow) and the missing vertical bar beneath the center of the Siemens star (marked with the purple arrow). The center of the reconstructed Siemens star is shown in Fig. 2a1 – the 50 nm spokes of the innermost ring are not properly resolved. In the central area of the scan, which was illuminated by both probes, the artifacts are strongly suppressed. The reconstruction of this scan, with uncoded, similar probes, has revealed two types of artifacts that originate from two different sources: missing or blurry features due to position errors of the scanning stage and ghost copies from other scan regions due to the ambiguity in disentangling the information from similar probing beams.

The first type of artifacts, namely, positioning errors, causing the missing bar (purple arrow in Fig. 2) and blurry 50 nm spokes of the innermost ring (Fig. 2a1), is a well-studied problem in single-beam ptychography, and position refinement^26^ can compensate for it. One of its drawbacks is that it creates more degrees of freedom in the solution of the inverse problem and significantly increases the total reconstruction time. In cases of severe position errors, the increase in time required for the reconstruction is especially high, as the search for the correct positions has to be performed over a large area. In parallel, the position errors complicate disentangling the diffraction signal from the different probes. As a result, artifacts resulting from the positioning errors show up in corresponding places of sample sub-regions for every beam, affecting the reconstructed image’s quality.

The use of coded probes greatly reduced the ambiguity in the diffraction signal from different regions of the sample affecting both types of artifacts which notably improved the reconstruction quality: Fig. 2b shows the reconstructed image of the Siemens star in the coded probe case. The comparison between Fig. 2a and b clearly shows that the artifacts are strongly reduced. Figure 2b1 illustrates the reconstruction quality: the 50 nm spokes of the innermost ring are well resolved.

**Figure 3:**
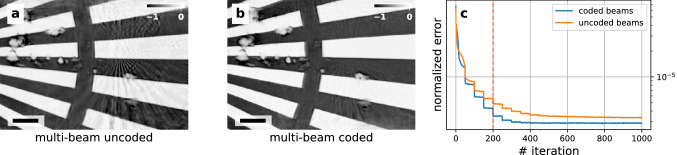
Cropped intermediate reconstruction results of the Siemens star after 200 iterations using (**a**) similar probes and (**b**) coded probes. The scale bars have a length of 1.5 μm. The grayscale indicates phase shift in radians. (**c**) Comparison of the evolution of the normalized error during the reconstruction of the scan of the Siemens star test sample with uncoded beams and coded beams. The sharp downward steps every 50 iterations are a result of the position correction being performed at these iterations.

Additionally to the improved image quality, we have observed an increase in the convergence speed of the ptychographic reconstruction. Figure 3 shows cropped intermediate reconstructed images from the scans with uncoded and coded probes after 200 iterations and the evolution of the normalized error of both reconstructions. The conspicuous difference in reconstruction quality can be seen when comparing between uncoded - Fig. 3a and coded - Fig. 3b probes: the scan with coded probes has no observable image artifacts while the one with uncoded probes has a ghost image of the Siemens Star center and blurry vertical bar.

**Figure 4:**
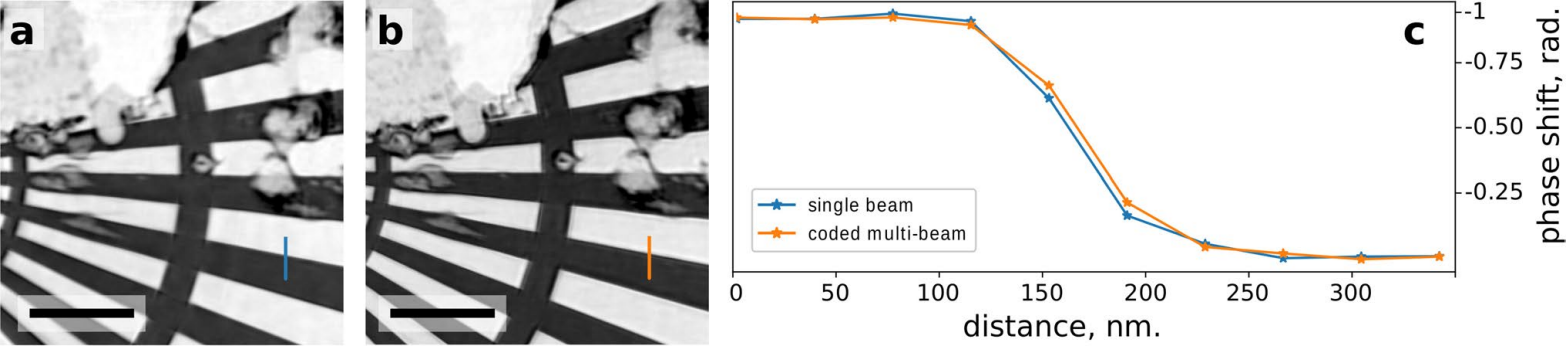
Evaluation of the resolution using line profiles. (**a**) Measurement with a single probe, (**b**) measurement with two coded probes, (**c**) corresponding profiles. The full width at half maximum value for the single beam profile is 64 nm and for the two beam profile 68 nm. The scale bars represent 3 nm.

The significantly improved reconstruction quality in the case of coded probes allows a direct comparison between multi-beam and single-beam ptychography. For this purpose we have performed a scan with a single beam with the same step size as for the two beam case. Figure 4a and b compare the quality of the reconstructed object using a single probe and using two coded probes, respectively. To quantitatively evaluate the resolution, two line profiles in Fig. 4 were fitted with an error function. The measured widths agree within 10 %, indicating that the resolution and the quality of the reconstruction for the multi-beam scan reach the level of a single-beam scan. Both scans were recorded with the same fluence, the single beam scan covered the area of 10 μm × 10 μm (*v* × *h*) with a speed of 18 μm^2^ min^−1^ and the two-beam scan covered the area of 10 μm × 52 μm (*v* × *h*) with a speed of 34 μm^2^ min^−1^.

### Microchip imaging

**Figure 5:**
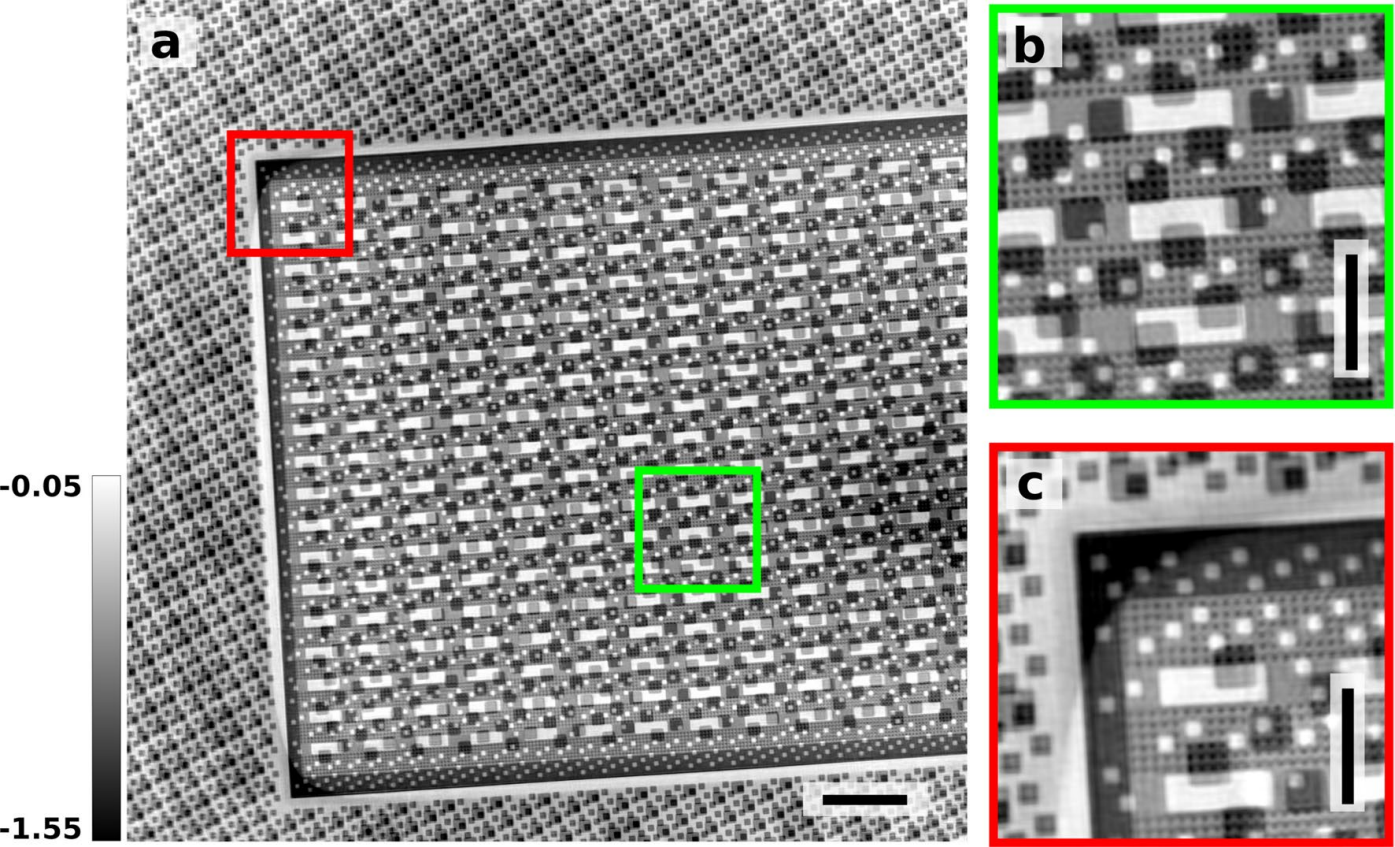
Reconstruction results from the microchip sample: (**a**) full view of the scanned area. The horizontal scale bar represents 8 μm. (**b**) enlarged view of the area marked by the green square. (**c**) enlarged view of the area marked by the red square. The vertical scale bars represent 3 μm. The grayscale indicates the phase shift in radians.

To apply the method to a real-world use case, we imaged a microchip structure produced by Infineon Technologies AG. We used three vertically-arranged coded probes (see Fig. 1d) to image an area of 82 μm × 80 μm with a similar overlap area between neighboring probes as in other measurements. The scan took around 1 hour and 14 minutes, covering the same area with a single probe would have taken roughly 2.7 times longer. Figure 5 shows the entire reconstructed image of the microchip and two enlarged areas to evaluate the reconstruction quality.

**Figure 6:**
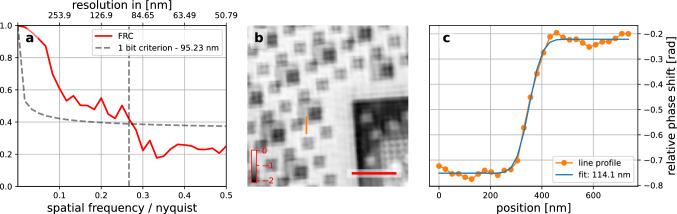
Resolution estimates of the images recorded of the microchip: (**a**) Fourier ring correlation of the reconstruction of two identical and consecutive scans, estimating a resolution of 95 nm. (**b**) edge feature on the phase image chosen for a line profile (orange line). The scale bar (red) has a length of 2 μm. (**c**) the extracted profile across the edge feature (orange) and the fit of an error function (blue) giving a resolution estimate of 114 nm.

To estimate the achieved resolution, the scan was repeated with identical scan parameters, field of view and coded probing beams. The two scans were reconstructed separately using identical reconstruction parameters. The two reconstructions were used to calculate the Fourier ring correlation (FRC)^27–29^ between them, estimating resolution of 95.23 nm (see Fig.6a). To verify that the two scans did not just reconstruct the same artifacts in the same place (something not caught by the FRC, as it measures the similarity of two images, but can not evaluate the correctness of either image), a line profile across a prominent edge (see Fig. 6b) was extracted and fitted with an error function, resulting in a resolution estimate of 114.1 nm (see Fig.6c).

## Discussion

The need to image large samples with the highest spatial resolution exists in many fields in science and industry and will increase with future needs in materials design. Due to the limited coherent flux at modern synchrotron radiation sources, multi-beam ptychography is a desirable solution. It enables the usage of the so far discarded incoherent fraction of the beam, increasing photon utilization in the experiment. While the coherent flux is greatly enhanced at the next generation synchrotron radiation sources with ultra-low emittance, the coherent fraction of hard X-rays at these sources will still be far from 100 %. Thus a scanning with multiple beams in ptychography will still be a highly desirable option.

Recent studies showed the potential to increase the acquisition speed or enlarge the scan area by covering the incident beam with closely packed lens arrays with a spacing smaller than the detector’s pixel size^21^. However, the improved photon utilization and scanning speed, as a result, came along with a decreased image quality. Due to the high ambiguity of the diffraction signal from several similar probes with a small separation and experimental uncertainties, the object reconstruction often contains artifacts and has a reduced resolution. These image artifacts may outweigh the benefit of parallel scanning and prevent the broader use of multi-beam scanning. In addition, the multi-beam reconstruction is more sensitive to unavoidable experimental uncertainties, such as positioning errors or mechanical vibrations.

In the current work, we have demonstrated the practical solution to the drawbacks in multi-beam ptychography by introducing diversity in the probes. We further reduced the probe separation to 26 μm, which positively affected the scanning speed as it allowed to perform a scan of a desired object’s area with fewer steps compared to measurements with larger probe separation. Using individually coded probes for multi-beam ptychography, we could image a larger area of the sample faster and with similar quality as compared to conventional ptychography. Compared to uncoded multi-beams, the unique topological charge of each beam accelerates the reconstruction and leads to faster convergence, here by a factor of three. To assess the reconstructed image quality, we have directly compared the results of the coded multi-beam and conventional single-beam ptychography, achieving a similar resolution. Furthermore, we used coded multi-beams to scan a microchip sample that contains regular and repeating structures^30^ that are challenging for multi-beam ptychography with similar probes. We imaged an area of 82 μm × 80 μm with no visible artifacts in just 1 hour and 14 minutes in a step-settle-expose scanning mode, while a similar scan using only a single beam would have taken nearly four hours. The proposed method enables rapid imaging of large sample areas with high resolution and high image quality, which is in high demand in many fields of science and industry^5,6^.

Combining multi-beam ptychography with continuous (fly)-scanning^31–33^ will allow to utilize most of the available X-ray beam and minimize any dead time during the scan. The continuous movement of the sample during each exposure can lead to smearing in the recorded diffraction patterns. The influence of this smearing on the reconstruction quality of multi-beam data is an open question.

Besides the smearing from fly-scanning, other factors could limit reconstruction quality. Mutual partial coherence between adjacent probing beams and thus interference effects between the signal from adjacent beams must be considered, or even better, avoided. By choosing a suitable sample–detector distance, coherence effects can be suppressed through *alias cloaking* or *auto correlation filtering*^17,34^. To avoid partial mutual coherence in the first place, it is possible to either design the X-ray optics aperture/spacing to match the transverse coherence length or, if the beamline allows for it, adjust the coherence length to fit the aperture. This could be done for example with slits to create a secondary source^35,36^ or by using prefocusing optics^37^.

It has been shown that the 3D printed X-ray optics can deteriorate and shrink in intense X-ray beams^38,39^. To counter this, the lenses could be designed with the shrinking in mind, or even pre-treated^40^ to avoid shrinking during the experiment. Another option is to exchange the optics frequently. As the manufacturing process is inexpensive, it is straightforward to print the same design multiple times. In previous experiments, we have experienced that 3D printed optics can be used for one week of beamtime without showing any deterioration^22,41^. The actual durability will vary from experiment to experiment and depend on the resist used and the energy and intensity of the photon beam.

The scan area was larger than the distance between adjacent probing beams in all multi-beam scans. Therefore, the scan regions of the individual beams overlap. Nevertheless, this overlap is not necessary for a successful reconstruction. To demonstrate this point, we increased the probe separation in a new reconstruction of the 2-beam scan of the Siemens star. This places the two scan regions so far apart that each scan region only interacts with one beam. The successful reconstruction is shown in the supplementary information (S1). Both sample regions were faithfully reconstructed without sharing any information in the sample plane. The reconstructed object is now split into two parts, and it requires stitching to obtain a single image of the whole sample. Overlapping the scan regions from adjacent probing beams avoids this post-processing as it forces the scan regions to match in the overlapping areas. This increases the data redundancy in the whole dataset, which also helps the reconstruction.

## Methods

### Scanning setup

The experimental studies were performed at the microprobe end-station of beamline P06^25^ of the synchrotron radiation source PETRA III at DESY in Hamburg, Germany. The experimental scheme is illustrated in Fig. 1a. We used an array of 3D-printed compound refractive lenses^22^ (A in Fig. 1a) to create multiple probes by focusing X-rays. A single lens has a square footprint of 25 µm × 20 μm and is 290 μm high. The single-lens aperture was illuminated by the X-ray beam with a flux of 2.2 × 10^7^ photons/sec. The lenses are placed in a 26 μm raster to avoid printing defects between neighboring lenses, leaving a 1 μm gap between them. Such an approach to forming probe arrays has a significant advantage as it leaves very little space not occupied by the optics and, as a result, allows higher photon utilization: (lens width/probe spacing)^2^ × 100%. Here, the experiments were performed with the probes arranged inline, leaving no gaps in the second direction. This yields a photon utilization of 50/51 × 100% = 98% for the two-beam case, and 75/77 × 100% = 97.4% for the three-beam case. These percentages are relative to the aperture of the lens stacks used and the spacing between them and thus represent the effectiveness of the arrangement of the X-ray optics. Assuming a homogeneous illumination of the lens stack, the number of photons utilized to probe the sample scales with the number of beams used, namely 196% for the two-beam case and 292.2% for the three-beam case.

Each lens comprises six single parabolic surfaces with an apex curvature of 2.5 μm. At the photon energy of 7 keV these lens stacks had a focal length of about 250 mm. Some of the lenses were equipped with phase plates creating beams with orbital angular momentum (OAM) with topological charges of + 1, − 1, or + 2, respectively^24^. This was realized by vortex phase plates with a smooth helical phase ramp. The phase plates and the lenses were printed together as one solid piece and are thus made from the same material. An individual phase plate covers the whole aperture of the lens it is placed on. The height of the phase plates spiral was designed to match a phase shift of $2\pi$ at 7 keV.

The upstream slits in front of the lens array were used to select different lens combinations. Background scattering from the lenses was reduced by placing a pinhole array between the lens array and the sample (B in Fig. 1a). The pinhole array was made from 100 μm thick platinum foil by laser ablation, with a pinhole size of 20 μm. As samples (C in Fig. 1a), a Siemens star resolution chart made by NTT-AT (XRESO-50HC) with smallest features of 50 nm and a microchip manufactured by Infineon Technologies AG was used. For the measurements with the Siemens star sample and microchip a photon energy of 7keV and 9keV were used, respectively.

The sample was placed into the approximate focal plane, and the diffracted beam was recorded with a single-photon counting, custom made in-vacuum Eiger 4M detector (pixel size 75 μm)^42^. The detector was mounted at the end of an evacuated flight tube to eliminate absorption and scattering in air^4,25^. The distance from the sample to the detector was 8200 mm for the Siemens star sample and 7100 mm for the microchip.

Figure 1b and c illustrate the uncoded and the coded lens combinations used in the measurements with the test sample, while (d) shows the coded three beam combination that was used to image the microchip. Prior to the multi-beam measurement, we verified the lenses by characterizing each beam individually using single-beam ptychography. Fig. 1e illustrates the complex wavefields of the probes in the object plane.

In the single beam measurement, the Siemens star was scanned in a grid of 21 × 21 = 441 (*v* × *h*) positions in 0.5 μm steps, with an exposure time of 0.5 s per point, covering a total area of 10 μm × 10 μm. The scan took around 5 minutes, including time for the movement of the scanning stage.

For the two-beam measurements with the Siemens star, the sample was scanned in a grid of 21 × 61 = 1281 (*v* × *h*) positions in 0.5 μm steps, with an exposure time of 0.5 s per point. Since the lenses were placed in a 26 μm raster, the scan areas of the two probes overlap on a 10 μm × 4 μm (*v* × *h*) wide area. The scan took around 15 minutes, including time for the movement of the scanning stage. With both probes the total area covered by the scan was 10 μm × 52 μm. The gain in scanning speed over the conventional single-beam scanning, in this case, was 1.9.

For the three beam measurement of the microchip, the sample was scanned in a grid of 61 × 161 = 9861 (*v* × *h*) positions in 0.5 μm steps with an exposure time of 0.2 s per point. The scan areas of the neighboring probes overlap on a 10 μm × 80 μm (*v* × *h*) wide area. With all three probes the total area covered by the scan was 82 μm × 80 μm. The gain in scanning speed over the conventional single-beam scanning, in this case, was 2.7. The total thickness of the imaged chip was 150 μm, while all visible features were in stacked embedded layers with a total thickness of 15 μm. The lateral size of the shown repeating features in these different layers varied between 2.5 μm and 250 nm.

### Reconstruction approach

For the reconstructions, the recorded far-field diffraction patterns were cropped to 512 × 512 pixels centered around the beam axis. For the single beam scan, the image was reconstructed with the standard ePIE algorithm^43^ for 500 iterations, followed by the modified coupled update^44^ for another 500 iterations. The pixel size in the reconstruction is 37.8 nm. To compensate for positioning errors of the scanning stage, the scan positions were numerically refined every 50 iterations to improve the reconstruction^26^.

In the two-beam scan, the experimental parameters were identical to the single beam scan, resulting in the same pixel size in the reconstruction. Following the same procedure as in Wittwer, et al.^21^, the image was reconstructed using 500 iterations of a modified multiprobe sPIE^45^ followed by 500 iterations of the modified coupled update^44^. We again used a local position refinement every 50 iterations.

For the three-beam scan of the microchip, the photon energy and detector distance were different, resulting in a pixel size of 25.4 nm in the reconstruction. Because we performed the experiment at a higher energy, the size of the probes illuminating the sample increased. We changed the reconstruction scheme for this scan: for the first 500 iterations we used the modified multiprobe sPIE^41,45^ with a 2 × 2 upsampling followed by 1500 iterations of the modified coupled update^44^. Also here, a local position refinement was used every 50 iterations.

## Supplementary Information

Supplementary Information.
