# Supplementary material for: Multi-beam X-ray ptychography using coded probes for rapid non-destructive high resolution imaging of extended samples
Source: Sci Rep. 2022 Apr 13;12:6203. doi: 10.1038/s41598-022-09466-5 (PMC9008058; doi:10.1038/s41598-022-09466-5)
Supplement: Supplementary file 1 — Supplementary Information. [file 41598_2022_9466_MOESM1_ESM.pdf]

SUPPLEMENTARY INFORMATION - MULTI-BEAM X-RAY PTYCHOGRAPHY USING CODED PROBES FOR RAPID NON-DESTRUCTIVE HIGH RESOLUTION IMAGING OF EXTENDED SAMPLES

MIKHAIL LYUBOMIRSKIY [1,*], FELIX WITTWER [1,2,**], MAIK KAHNT [3], FRIEDER KOCH [4,5+], ADAM KUBEC [4,6+], KEN VIDAR FALCH [1], JAN GARREVOET [1], MARTIN SEYRICH [1,2], CHRISTIAN DAVID [4], AND CHRISTIAN G. SCHROER [1,2,7]

[1] CXNS – Center for X-ray and Nano Science, Deutsches Elektronen-Synchrotron DESY, Notkestr. 85, 22607 Hamburg, Germany
[2] Department Physik, Universität Hamburg, Luruper Chaussee 149, 22761 Hamburg, Germany
[3] MAX IV Laboratory, Lund University, Box 118, 221 00 Lund, Sweden
[4] Paul-Scherrer-Institut (PSI), Forschungsstr. 111, 5232 Villigen-PSI, Switzerland
[5] GSI Helmholtzzentrum für Schwerionenforschung GmbH, Planckstr. 1, 64291, Darmstadt, Germany
[6] XRnanotech GmbH, Forschungsstr. 111, ODRA 117, 5232 Villigen-PSI, Switzerland
[7] Helmholtz Imaging Platform, Deutsches Elektronen-Synchrotron DESY, Notkestr. 85, 22607 Hamburg, Germany
* mikhail.lyubomirskiy@desy.de
** Current address: NERSC, Lawrence Berkeley National Laboratory, Berkeley, California 94720, USA
+ these authors contributed equally to this work

S 1 shows the comparison of reconstructions of the same data set (coded beams) with different probe spacing. Overlapping of the scanned areas is not required for successful reconstruction. Nevertheless, the presence of overlap helps with obtaining the final object image, avoiding post-processing.

S 2 shows the comparison of the structure between both uncoded beams (left) and both coded beams (right). Both uncoded beams were created by two lens stacks with identical design parameters. Still, there are minute differences in the printed result. These minute differences in the optics creating the multiple beams will eventually result in slight differences in the created probing beams. This is true for all multiple-beam experiments, as each beam is created by its own optical element. Nevertheless, these minute differences are not the major factor that helps disentangle the information from different probes in the multi-beam reconstruction. Great differences in the phase distribution and significant differences in the intensity minima and maxima of the coded probing beams will result in different places for the interference maxima and minima on the detector. Ptychographic reconstruction algorithms are far more sensitive to this richness in otherwise unexplainable intensity maxima and minima (speckles) and far less sensitive to minute changes in relative intensities of the same speckles.

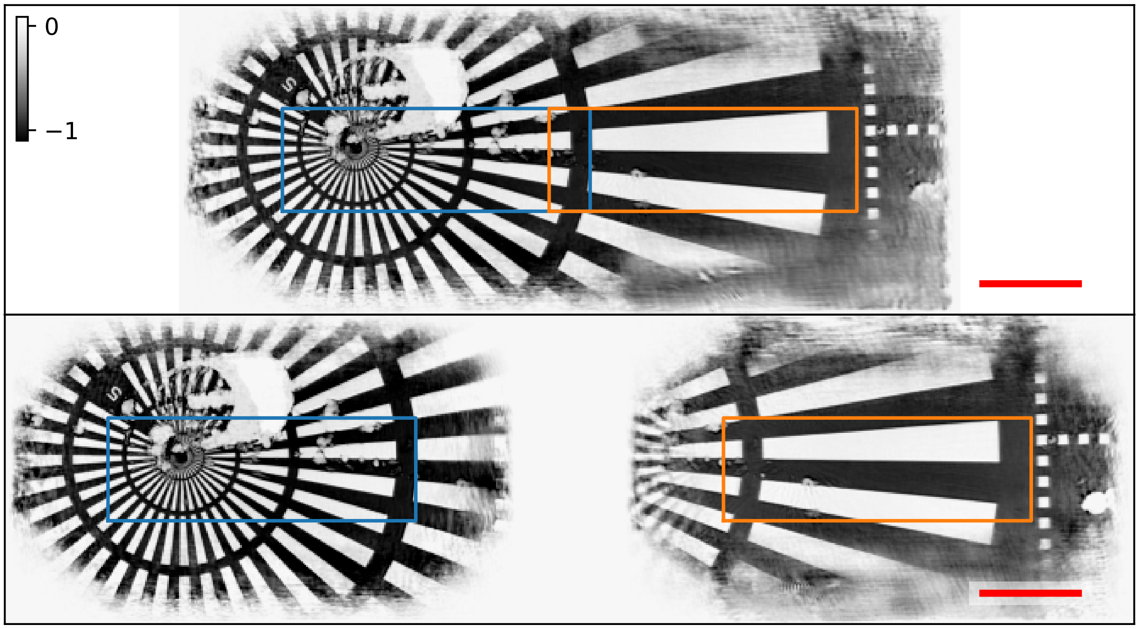

S 1. Multi beam reconstructions of the same data set with the correct probe spacing (top) and too large probing spacing (bottom). The blue and orange rectangles mark the areas scanned by the center of the probing beams. The scale bars (red) have a length of 10 µm. The grayscale indicates the relative phase shift in radians.

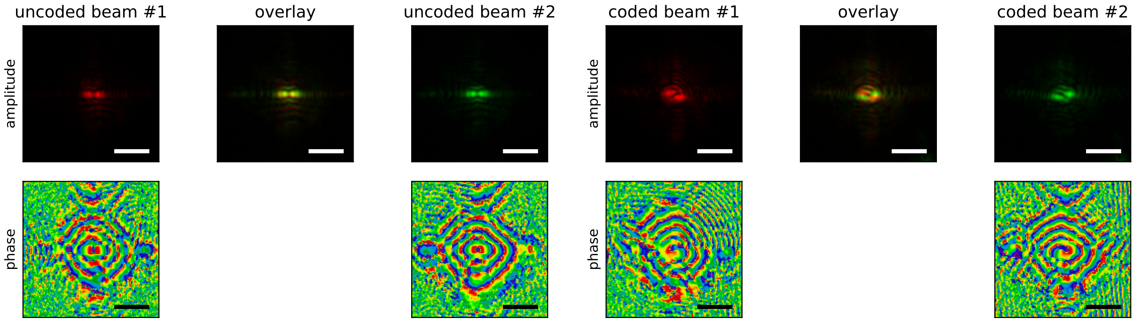

S 2. Comparison of the structure both uncoded beams (left) and both coded beams (right) of the siemens star reconstruction. Where the uncoded beams are very similar to each other in amplitude and in phase, the coded beams vary in where the intensity minima and maxima are located as well as the direction of the phase spiral. All scale bars have a length of 5 µm.
